# Analysis of Acupoints Combination for Cancer-Related Anorexia Based on Association Rule Mining

**DOI:** 10.1155/2022/4251458

**Published:** 2022-10-18

**Authors:** Yang Tang, Yanju Liang, Xing Wang, Li Deng

**Affiliations:** ^1^Department of Oncology, The Affiliated TCM Hospital of Guangzhou Medical University, Guangzhou 510000, China; ^2^The First Clinical College of Guangzhou University of Chinese Medicine, Guangzhou 510000, China; ^3^Department of Acupuncture, The Affiliated TCM Hospital of Guangzhou Medical University, Guangzhou 510000, China

## Abstract

We investigated the acupoint selection regulations and workable core acupoint combinations in cancer-related anorexia (CA) treatment. The Apriori algorithm, complemented by the FP-growth algorithm, was used to mine association rules based on retrieved randomized control trials (RCTs) and clinical control trials (CCTs). We searched the following databases for acupuncture treatment regimens for CA: PubMed, Embase, Cochrane Central, Web of Science, WanFang Data, VIP, China Journal Full-Text Database (CNKI), and SinoMed (CBM). We extracted acupoints prescriptions from the 27 enrolled RCTs and CCTs for analysis. There have been 38 acupoints refined from 27 prescriptions. The pinnacle three regularly chosen acupoints were Zusanli (ST36), Zhongwan (RN12), and Sanyinjiao (SP6). We investigated 10 association rules, and the consequences confirmed that {RN4} ≥ {RN12}, {PC6} ≥ {ST36}, {RN12, SP6} ≥ {RN4}, {HT7} ≥ {RN12}, and {DU20} ≥ {RN12} had been the most frequent associated rules in the adoption literature. Zusanli (ST36), Sanyinjiao (SP6), Guanyuan (RN4), Zhongwan (RN12), Neiguan (PC6), Shenmen (HT7), and Baihui (DU20) would be regarded as acupuncture prescriptions in the treatment of CA.

## 1. Introduction

According to statistics, there were 23.6 million new cancer cases in 2019 (17.2 million without nonmelanoma skin cancers), an estimated 10.0 million cancer deaths, and 250 million disability-adjusted life years associated with cancer [1]. In addition to cancer-related morbidity and mortality, patients' life quality has been severely affected during treatment and recovery [2]. Cancer-associated anorexia (CA), defined as loss of appetite, early satiety, or changes in food taste or smell, negatively affects cancer patients' health-related quality of life [3]. CA is widespread amongst most oncological patients, affecting 30–80% of cancer patients referred to palliative care [4]. Anticancer drugs [5], radiation [6], pain, depression, alimentary tract obstruction, decrease in taste sensitivity, slowing of gastric emptying, disturbance of appetite-satiety centers, and tumor-released cytokines result in CA's occurrence [7–9]. Pharmacotherapeutic agents such as corticosteroids, megestrol acetate, cyproheptadine, hydrazine sulfate, and dronabinol are used in treating this condition [10]. Aside from their limited therapeutic efficacy, these therapies also cause adverse effects. As for the guidelines from the National Comprehensive Cancer Network (NCCN), A range of nonpharmacologic approaches, medications, and assessments is recommended [10]. During cancer treatment, anorexia remains one of the most common and troubling clinical problems [11, 12]. It is necessary to develop alternative therapy for CA.

Acupuncture (AP), as a worldwide alternative medical therapy, has raised public concern for its variable effectiveness in improving symptoms among cancer patients [13, 14]. According to clinical trials, AP has been proven to reduce acute vomiting caused by chemotherapy, reduce insomnia, and relieve pancreatic cancer pain [15]. The underlying therapeutic mechanisms of acupuncture in alleviating CA include anti-inflammatory effects, appetite stimulation, fatigue alleviation, and stimulation of ghrelin secretion [16]. The selection of points and their compatibility applications are vital for the efficacy of acupuncture [17]. The selecting and combining of acupoints are built on the syndrome differentiation and the theories of the Meridian-Viscera Association theory [18]. However, the standard of acupoints in CA treatment does not reach a consensus.

Data mining strategies have recently become a predominant field of research with broad applications in acupuncture and Chinese medicine, treating Alzheimer's disease [19] and vascular dementia [20]. Previous research provides evidence for the choice and combination of acupuncture points based on literature mining. Data mining involves several methods, such as associations, correlations, classifications, and clustering [21]. In clinical practice, acupuncture therapy involves treating multiple acupoints simultaneously. Hence, association rule analysis helps explore the basic principles that identify the set of items or attributes that occur together.

Apriori is a standard association rule mining method used to find the frequent itemsets in data sets. Frequent itemset mining refers to determining the items that often occur together in a data set [22]. Therefore, the Apriori algorithm helps find regularities in data by analyzing association rules between acupoints. Apriori uses particular metrics to understand associations' strengths: support, confidence, and lift. On the other hand, the FP-growth algorithm is an improvement over the Apriori algorithm, analyzing the most frequent patterns in a dataset to present them as an association tree [23]. Like the Apriori algorithm, it has basic metrics, support, confidence, lift, and expected confidence for evaluating frequent patterns. Finding such frequent sets is suitable for acupoint clinical decision-making [24, 25].

In this paper, we aim to identify the possible core combination of acupoints appropriate for the treatment of CA by analyzing retrieved RCTs and CCTs using the Apriori algorithm-based association rule. Afterward, the results were compared to those obtained with the FP-growth algorithm. We are further grasping the acupoint selection rules and core acupoint pairs of acupuncture in the treatment of CA to provide a reference for clinical medicine.

## 2. Materials and Methods

### 2.1. Literature Search

As of January 2022, we used the following eight databases for original research articles: China Journal Full-Text Database (CNKI), VIP, WanFang Data, and SinoMed (CBM), as well as PubMed, Cochrane Central, Embase, and Web of Science. The retrieval strategies have used a combination of medical subject headings (MeSH) and keywords. The MeSH and keywords incorporate “Anorexia” AND “neoplasms,” AND “acupuncture therapy,” OR “electroacupuncture,” OR “moxibustion,” OR “acupoint embedding,” OR “acupoint injection.” Chinese characters with equal means had been used for literature retrieval in Chinese databases. For different databases, we adjusted the search terms accordingly. There were no language restrictions. We searched the ClinicalTrials.gov registry (https://clinicaltrials.gov/) for unpublished studies.

### 2.2. Inclusion Criteria

The inclusion criteria were as follows:

1. Research on acupuncture treatment for CA included randomized controlled trials (RCTs) and clinical control trials (CCTs)
2. The included literature must have a clear prescription for acupuncture
3. Literature on acupuncture and moxibustion can play a fundamental and adjunctive therapeutic role (such as a combination of acupuncture and medicine)
4. The treatment methods were limited to acupuncture, electroacupuncture, moxibustion, acupoint embedding, acupoint injection, and comprehensive application
5. An enrolled study must measure appetite change or the Visual Analog Scale (VAS) for anorexia

### 2.3. Exclusion Criteria

The exclusion criteria were as follows:

1. The literature does not meet any of the criteria above
2. Auricular points, animal experiments, cross-sectional studies, comments, cohort studies, and repeated publications were excluded

### 2.4. Quality Assessment

The included trials were assessed using a Cochrane risk assessment tool. Five domains of bias are present in the device: randomization process, deviations from intended interventions, missing outcome data, measurement of the outcome, and selection of the reported result. Risk categories included “high risk,” “low risk,” and “some concerns.” Two independent reviewers assessed the risk of bias in each study at the title, abstract, and full-text levels.

### 2.5. Data Analysis

Each acupoint was extracted based on distinctive Chinese syndrome differentiation to analyze associations. The name and location of acupoints followed the World Health Organization standard. First, we figured up the Cumulative frequency of acupoints in the cure of CA. We calculated point frequency by dividing the number of covered studies by the number of points. After that, we performed an Apriori association rule analysis to determine the frequently used acupoint combinations and the correlation between distinctive acupoints. Lastly, we summarized the clinical decision-making of acupuncture in CA treatment.

R version 4.2.1 was used to conduct Apriori association rule analysis and plot charts. A dataset consists of 33 columns, each containing a unique formula. We then fitted the dataset using the R package “arules” and visualized the charts using the R package “arulesViz.” For comparison, A frequent pattern growth algorithm was conducted using Python's open-source package, “PyFpgrowth.”

In apriori algorithm-based association rules, each listing item has an antecedent (left-hand-side, LHS) and a consequence (right-hand-side, RHS). Support, confidence, expected confidence, and lift are the four kernel values that make up an association rule analysis. Support is calculated as the number of occurrences divided by the total number of records for an item set. In an itemset, support is used to determine the frequency with which item sets occur. When we discover rules, we focus on the high-frequency itemsets. Confidence is the probability that *Y* will happen in the presence of *X*, and lift is related to mined association rule value. The expected confidence showed the likelihood of the consequences when the antecedent was independent of the consequent. Users must combine support and confidence factors multiple times to discover significant association rules. Although thresholds were selected in different cases, their selection had subtle ambiguities. A high parameter threshold would result in the loss of certain meaningful information.

We studied the top 10 commonly used association rules with 24% and 75% thresholds for support degree and confidence. In addition, we reported association rules based on their descending support, confidence, and lift values. Twenty-one commonly used acupoints mentioned in the RCTs and CCTs were clustered by clustering algorithm in SPSS software (IBM SPSS 23.0, SPSS Inc) and were divided into 4–10 classes by system clustering.

## 3. Results

### 3.1. Eligible Studies and Study Characteristics

**Figure 1:**
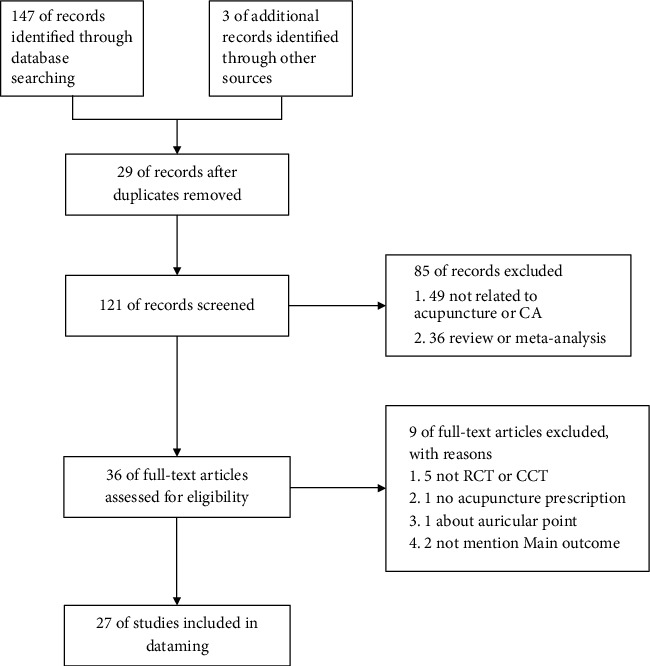
Flowchart of literature screening.

We screened 150 related articles and removed 29 duplicates. Based on titles and abstracts, we screened 121 final research articles, leaving 36 likely to be eligible. Following the full-text reading, 27 articles were finally adopted [26–32]; some are In Chinese. The flow of this study is presented in Figure 1. The median duration of therapy was two weeks, and the median number of acupuncture points was four.

### 3.2. Risk of Bias Assessment

**Figure 2:**
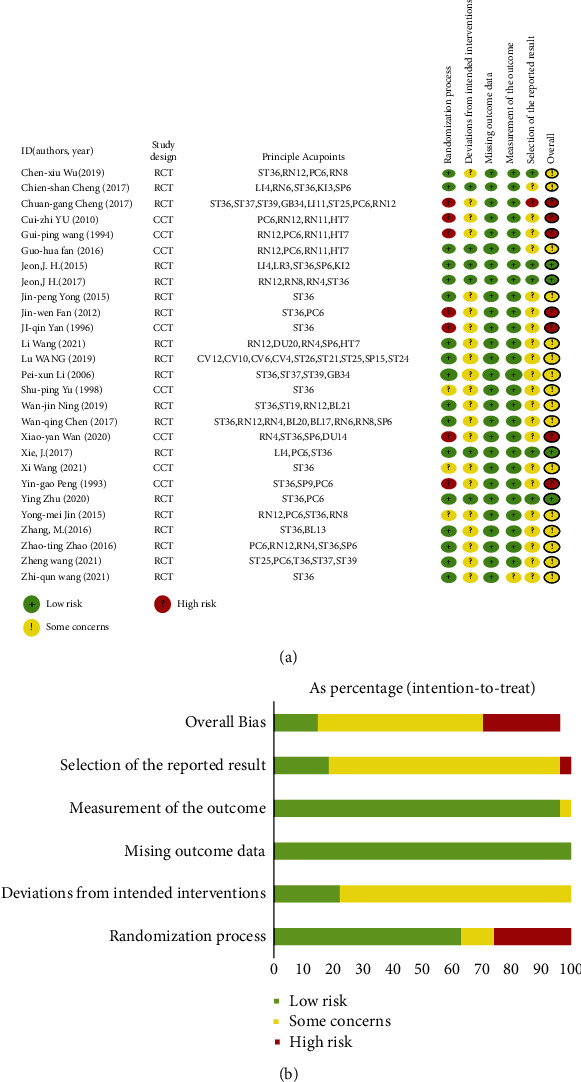
Risk of bias assessment: (a) summary of risk of bias items for each included study; (b) graph of bias risk.

(See Figure 2) shows bias assessments for the 27 included studies. In most studies, deviation from the intended intervention and selection of the reported result was uncertain. The 27 RCTs showed variable overall quality. The reason may be due to one of the following factors. Seventeen out of 27 RCTs and CCTs had apparent descriptions of the randomized process. Among the retrieved studies, only three used patient blinding with sham acupuncture, and ten did not compare CA symptoms severity at baseline.

### 3.3. Distribution of the Acupoint

**Figure 3:**
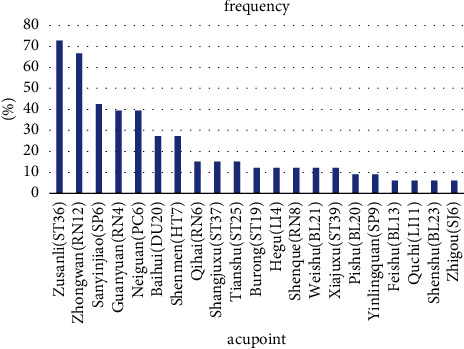
The frequency and proportion of the top 10 used acupoints for the treatment of CA in the retrieved studies.

Among the 27 retrieved studies, 38 acupoints were identified. The top 10 regularly chosen acupoints were Zusanli (ST36), Zhongwan (RN12), Sanyinjiao (SP6), Guanyuan (RN4), Neiguan (PC6), Baihui (DU20), Shenmen (HT7), Qihai (RN6), Shangjuxu (ST37), and Tianshu (ST25).  Figure 3 shows the distribution details of acupoint frequency.

### 3.4. Apriori Algorithm-Based Association Rule Analysis of Acupoint Combinations

Our study evaluated the ten frequently used association rules, and the minimum threshold was established as a support degree of 24% and confidence of 75%.

**Figure 4:**
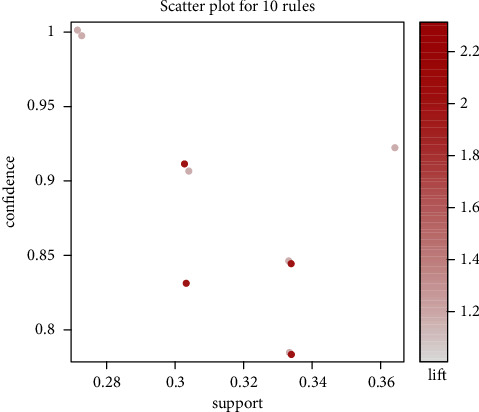
The scatter plot for the ten association rules was determined from 27 RCTs and CCTs of acupuncture treatment for CA using an Apriori algorithm.

We examined ten association rules based on 33 acupoint formulas to treat CA. In Figure 4, each association rule was represented by a scatter plot with its support and confidence axes. Each association rule was colored based on its metric value and lift. Results showed that ten association rules had lift values ≥ 1. Therefore, acupoints selected with their respective antecedents were statistically significantly more likely to be included in the same formula than just the consequent acupoint alone. All ten association rules had metric values ≥ 0.75 for confidence. Consequently, each rule has a relatively high probability of selecting the consequent acupoint when antecedents are chosen. Support values ranged from 0.28 to 0.36, representing the frequency of occurrence for each antecedent acupoint. This result suggests the possibility of a core set of acupoints for treating CA.  Table 1 lists the top 10 Apriori algorithm-based association rules of acupoints.

**Figure 5:**
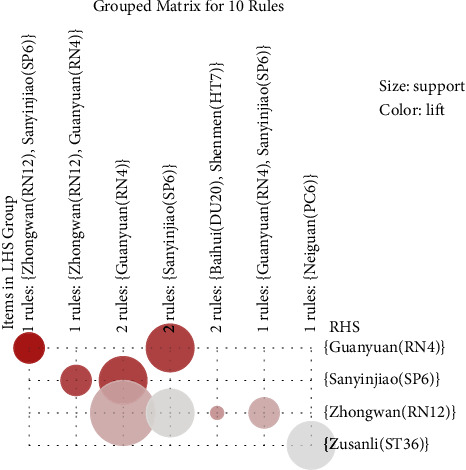
Grouped matrix for ten association rules.

**Figure 6:**
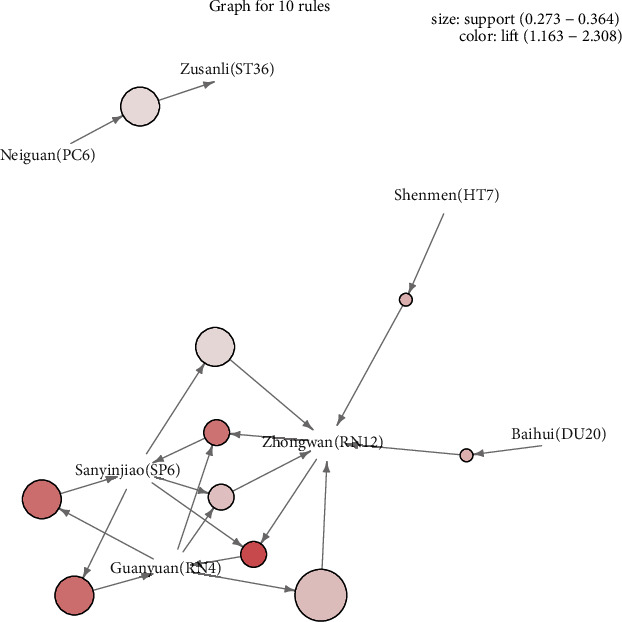
Graph of association rules obtained in the 27 RCTs and CCTs.

Figure 5 illustrates the general distribution of association rules using a grouped matrix diagram. On the horizontal ordinate, seven clusters were shown, and on the vertical ordinate, the acupoints were produced by those clusters (rules). For a group, circles with darker colors indicate a greater degree of lift, and circles with larger sizes indicate more significant support values. As shown in Figure 6, 10 of the 33 acupoint formulas are associated with ten association rules. The most common use of Zhongwan (RN12), Guanyuan (RN4), and Sanyinjiao (SP6) were in conjunction with another acupoint. Neiguan (PC6) and Zusanli (ST36) were commonly used Acupoint combinations.

### 3.5. Frequent Pattern Mining for CA with Frequent Pattern Growth Algorithm

The 27 RCTs and CCTs based on the frequent pattern growth algorithm are summarized in Table 2, and their results are consistent with Figure 6. According to the frequent item set, we can obtain the commonly used acupuncture combination: #2 (Baihui (DU20), Zhongwan (RN12)}, #4 {Shenmen (HT7), Zhongwan (RN12)}, #6 {Guanyuan (RN4), Sanyinjiao (SP6), Zhongwan (RN12)}, #8 {Neiguan (PC6), Zhongwan (RN12)}, #9{Neiguan (PC6), Zusanli (ST36)}, #11 { Sanyinjiao (SP6), Zhongwan (RN12)}, and #13 {Zhongwan (RN12), Zusanli (ST36)} (Table 2). Likewise, the result of frequent patterns is consistent with Frequent pattern growth algorithm-based association rules (Table 3). Due to the low lift values associated with PC6 and ST36, these rules were not selected in the association rules.

### 3.6. Cluster Analysis Results

**Figure 7:**
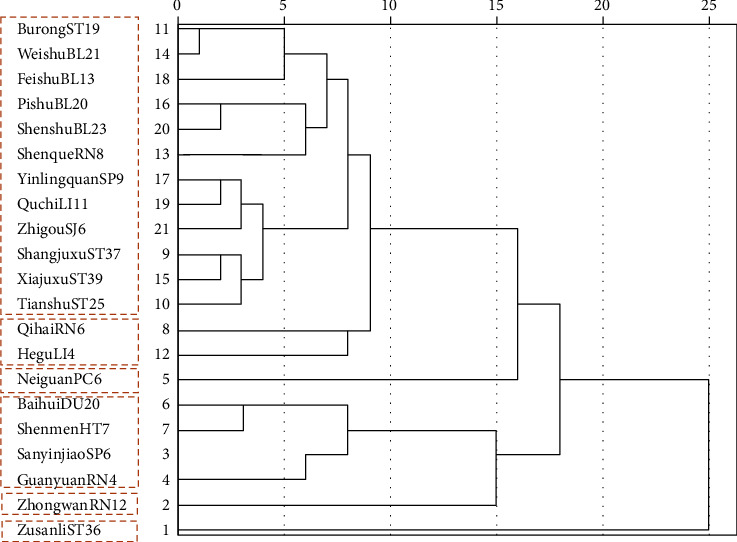
Cluster analysis plot.

SPSS23 software was used to cluster and analyze the acupoints, and a dendrogram was obtained (Figure 7). The dendrogram showed that acupoints were divided into three categories, among which subcategories could be further divided. According to the results of cluster analysis, six clusters of core acupoints could be obtained: Zusanli (ST36), Zhongwan (RN12), Baihui (DU20)-Shenmen (HT7)-Sanyinjiao (SP6)-Guanyuan (RN4), Neiguan (PC6), Qihai (RN6)-Hegu (LI4), and Burong (ST19)-WeishuBL (21)-Feishu (BL13)-Pishu (BL20)-Shenshu (BL23)-Shenque (RN8)-Yinlingquan (SP9)-Quchi (LI11)-Zhigou (SJ6)-Shangjuxu (ST37)-Xiajuxu (ST39)-Tianshu (ST25).

## 4. Discussion

**Figure 8:**
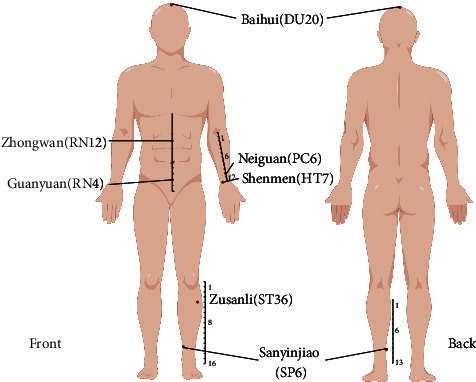
Acupoint locations derived from association rules obtained from 27 RCTs and CCTs.

This study analyzed the typical patterns of acupuncture point resolution for CA using data mining strategies. Our study indicated that Zusanli (ST36), Zhongwan (RN12), Guanyuan (RN4), Neiguan (PC6), Sanyinjiao (SP6), Shenmen (HT7), and Baihui (DU20) as the primary acupoint combinations (Figure 8). RCTs and CCTs of acupuncture for CA validated by Apriori association-mining indicate that the acupoint combination reduces loss of appetite symptoms in patients suffering from CA. It is more effective to combine acupoints than to use just one acupoint.

The etiology of CA includes treatment-related factors and patient-related factors. Treatment-related factors for CA are caused by chemotherapy [33, 34], radiotherapy [34, 35], and immunotherapy [34, 36]. The patient-related factors of CA are complex. Pain, depression, taste disorders, delayed emptying, dysregulation of the hypothalamic appetite signaling system, and cytokines released by tumors are considered essential factors in CA's pathogenesis.

The arcuate nucleus, a group of neurons in the hypothalamus, dominates appetite regulation. In anorexia, two neurons in the arcuate nucleus of the hypothalamus regulate appetite [37]. Neuronal feed intake stimulators coexpresses neuropeptide Y (NPY) and agouti gene-related protein (AgRP), as well as appetite suppressor neurons, coexpress pro-opiomelanocortin (POMC), and cocaine and amphetamine-regulated transcript (CART) [38]. Feed intake stimulator neurons and appetite suppressor neurons constitute two different complementary pathways. These neurons form two ways: one that stimulates energy intake and the other that inhibits it. Studies have shown that these pathways play an essential role in CA [39]. These pathways are closely related to the interaction of multiple mediators.

Tumor cells and surrounding tissues secrete a variety of proinflammatory factors (such as IL-1 and 6 and TNF) to promote the expression of proinflammatory factors in the hypothalamus, leading to the inactivation of NPY/AgRP neurons and activation of POMC/CART neurons, resulting in anorexia and other symptoms [40]. The anorexic peptide leptin regulates the appetite of hypothalamic appetite centers by inhibiting food intake and stimulating energy consumption [41]. Leptin interacts with a variety of neuropeptidergic molecules in the hypothalamus. When leptin levels are low, NPY/AGRP neurons are activated, and POMC/CART expression is inhibited, resulting in improved appetite, reduced energy expenditure, and increased food intake. Serotonin, a neurotransmitter synthesized from tryptophan, is often thought to play a vital role in vomiting, and 5-HT receptor 3 (5-HT3R) antagonists (such as ondansetron) are one of the most widely used antiemetics [42]. Ghrelin, an endogenous appetite-stimulating hormone, can stimulate food intake by promoting the expression of NPY/AgRP [43].

The possible fundamental mechanisms regulating levels of hormones and neurotransmitters may explain the success of the core acupoint combination in patients with CA. Studies have shown that stimulation of acupoint Zusanli(ST36) in rats could reduce the gastrointestinal hormone levels of ghrelin (GHRL), peptide YY(PYY), and glucagon-like peptide-1 (GLP-1), contributing to the amelioration of dyspepsia induced by cisplatin [41]. It has been found that stimulation of Zusanli (ST36) and Zhongwan (RN12) together can have antagonistic or synergistic effects on physiological and biochemical indicators, including gastric motility, ghrelin (GHRL), gastrin, and growth factor 1 [36]. The simulation of Zhongwan (RN12) in rats showed a significant antiemetic effect. The mechanism might be related to reducing 5-HT levels in the duodenum and suppressing the expression of c-Fos in the nucleus tractus solitaries (NTS) [44]. Electroacupuncture at Neiguan (PC 6) and Jianshi (PC 5) may reduce chemotherapy-induced nausea and vomiting, possibly by lowering 5-HT3 and dopamine levels [45].

Since an acupoint combination treatment efficacy is higher than a single acupoint [46, 47], in clinical application, acupoints are used in conjunction with each other [48]. ZHANG X. et al. reported that in patients with liver cancer, transcutaneous electrical stimulation of Neiguan (PC 6) and Jianshi (PC 5) improves gastrointestinal disturbances caused by transcatheter arterial chemoembolization [49]. An RCT that enrolled 30 patients with gastrointestinal cancer demonstrated that the combination acupuncture points of ST36, SP6, PC6, HT7, RN12, and so on may benefit some patients with GI cancer cachexia by normalizing metabolic dysregulation [32].

Acupoint combinations based on distal-proximal points, exterior-interior points, and regulating spirit points are the most common strategies for selecting acupuncture points. The proximal points are located near the ailment site, whereas the distal points are associated with the meridian that links the ailment site to the affected meridian. Distal points are the critical rule of point choice primarily based on the meridian theory, “The meridians pass by and the main indications reach.” The historically described meridian system provides a sound empirical reference system in sound distal from ailment sites. Acupuncture or heat moxibustion stimulates specific points in the body to balance energy flow through particular channels [50]. This energy flow correlates with specific organ systems and modulatory functions when enabled. Combined with the pathogenesis of CA and the motion traits eight acupuncture on the body, the possible mechanism of acupuncture in improving CA may be regulating the hypothalamus-pituitary-target gland axis [51].

Even though studies were exploring the Clinical curative effect of acupuncture in the remedy of CA, they, with fewer databases, did not pick out higher quality articles, such as RCT, and did not specify the association analysis. Our analysis of the association was based on an Apriori algorithm. According to these findings, CA point selection is associated with hypothalamus-pituitary-target gland axis regulation. We expect a data-based approach to go deep into our medical understanding concerning CA management using acupuncture.

### 4.1. Limitation

Although we suggested the core acupoint combination on CA, our study has several limitations. First, there is a high risk of bias in most RCTs and CCTs included in our study; most lack a careful follow-up. Second, many factors affect acupuncture's effect, including the depth of acupuncture, manipulation, retention time, treatment frequency, and course of treatment. However, these factors were not analyzed in this study. Third, since the acupoint combination's mechanism of action is still unclear, more basic and clinical studies are needed to evaluate it thoroughly.

## 5. Conclusion

Based on data mining analysis from RCTs and CCTs, the combination acupoints of Zusanli (ST36), Zhongwan (RN12), Guanyuan (RN4) and Neiguan (PC6), Sanyinjiao (SP6), Shenmen (HT7), Baihui (DU20) are potential acupuncture treatments for CA. Our analysis recommends the core acupoint combination for further primary mechanism research studies, clinical trials, and treatment strategies.

## Figures and Tables

**Table 1:** Top 10 Apriori algorithm-based association rules of acupoints.

No.	Association rules			Support	Confidence	Expected confidence	Lift
1	{Guanyuan (RN4)}	≥	{Zhongwan (RN12)}	0.364	0.923	0.67	1.385
2	{Neiguan (PC6)}	≥	{Zusanli (ST36)}	0.333	0.846	0.72	1.163
3	{Guanyuan (RN4)}	≥	{Sanyinjiao (SP6)}	0.333	0.846	0.39	1.995
4	{Sanyinjiao (SP6)}	≥	{Guanyuan (RN4)}	0.333	0.786	0.39	1.995
5	{Sanyinjiao (SP6)}	≥	{Zhongwan (RN12)}	0.333	0.786	0.67	1.179
6	{Guanyuan (RN4), Sanyinjiao (SP6)}	≥	{Zhongwan (RN12)}	0.303	0.909	0.67	1.364
7	{Guanyuan (RN4), Zhongwan (RN12)}	≥	{Sanyinjiao (SP6)}	0.303	0.833	0.39	1.964
8	{Sanyinjiao (SP6), Zhongwan (RN12)}	≥	{Guanyuan (RN4)}	0.303	0.909	0.39	2.308
9	{Shenmen (HT7)}	≥	{Zhongwan (RN12)}	0.273	1.000	0.67	1.500
10	{Baihui (DU20)}	≥	{Zhongwan (RN12)}	0.273	1.000	0.67	1.500

**Table 2:** Frequent patterns of acupoints used in the 27 RCTs and CCTs on acupuncture treatment for CA.

NO.	Frequent pattern	Counts
1	Baihui (DU20)	9
2	Baihui (DU20), Zhongwan (RN12)	9
3	Shenmen (HT7)	9
4	Shenmen (HT7), Zhongwan (RN12)	9
5	Guanyuan (RN4), Sanyinjiao (SP6)	11
6	Guanyuan (RN4), Sanyinjiao (SP6), Zhongwan (RN12)	10
7	Guanyuan (RN4), Zhongwan (RN12)	12
8	Neiguan (PC6), Zhongwan (RN12)	9
9	Neiguan (PC6), Zusanli (ST36)	11
10	Sanyinjiao (SP6)	14
11	Sanyinjiao (SP6), Zhongwan (RN12)	11
12	Zhongwan (RN12)	22
13	Zhongwan (RN12), Zusanli (ST36)	13
14	Zusanli (ST36)	24

**Table 3:** Frequent pattern growth algorithm-based association rules.

NO.	LHS	RHS	Confidence
1	Baihui (DU20)	Zhongwan (RN12)	1
2	Shenmen (HT7)	Zhongwan (RN12)	1
3	Sanyinjiao (SP6)	Zhongwan (RN12)	0.79
4	Guanyuan (RN4) Sanyinjiao (SP6)	Zhongwan (RN12)	0.91
5	Guanyuan (RN4) Zhongwan (RN12)	Sanyinjiao (SP6)	0.83
6	Sanyinjiao (SP6) Zhongwan (RN12)	Guanyuan (RN4)	0.91

## Data Availability

The data utilized to support the findings of this study are included within the article.
